# Correction: *Ex-vivo* cultured human corneoscleral segment model to study the effects of glaucoma factors on trabecular meshwork

**DOI:** 10.1371/journal.pone.0238408

**Published:** 2020-08-25

**Authors:** Ramesh B. Kasetti, Pinkal D. Patel, Prabhavathi Maddineni, Gulab S. Zode

Fig 4, Fig 5 and Fig 6 are incorrect–panels Fig 4C, Fig 5C and Fig 6D are incomplete. The authors have provided the corrected versions here.

**Fig 4:**
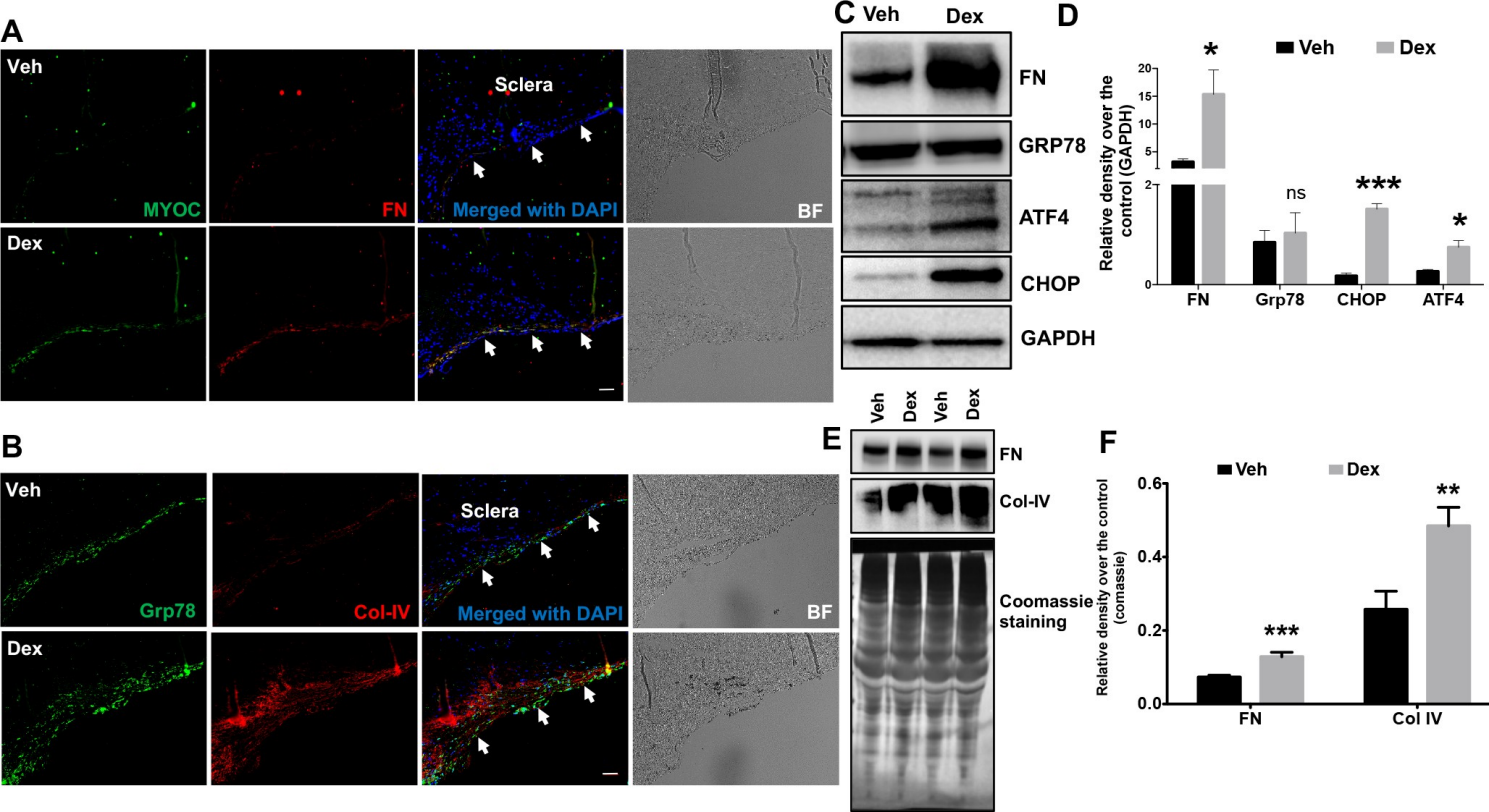
Increased ECM accumulation and ER stress induction in the TM of Dex-treated cultured corneoscleral segments. **A**) Immunostaining for myocilin and fibronectin, **B)** GRP78 (ER stress marker) and collagen IV in quadrants cultured for 7 days treated with the vehicle (0.1% ethanol) and Dex (100nM). Dex treatment prominently increased myocilin, fibronectin, collagen IV and GRP78 staining in the TM region. (n = 4 biological replicates, scale bar is 50μm). Western blot and densitometric analysis for FN (ECM marker) and ATF4, CHOP and GRP78 (ER stress markers) in TM tissue lysates (**C-D**) of vehicle- and Dex- (100nM) treated cultured corneoscleral segments. Dex treatment led to a significant increase in the ECM marker, FN (n = 3 biological replicates) and ER stress markers CHOP and ATF4 but not GRP78 (n = 4 biological replicates). Note that densitometric analysis included only Dex responders. Similarly, conditioned medium (**E-F**) from Dex-treated corneoscleral segments showed a significant increase in ECM proteins FN and Col IV (n = 8); unpaired t-test, *P<0.05, **P<0.01, ***P<0.001). Arrows indicate the TM region.

**Fig 5:**
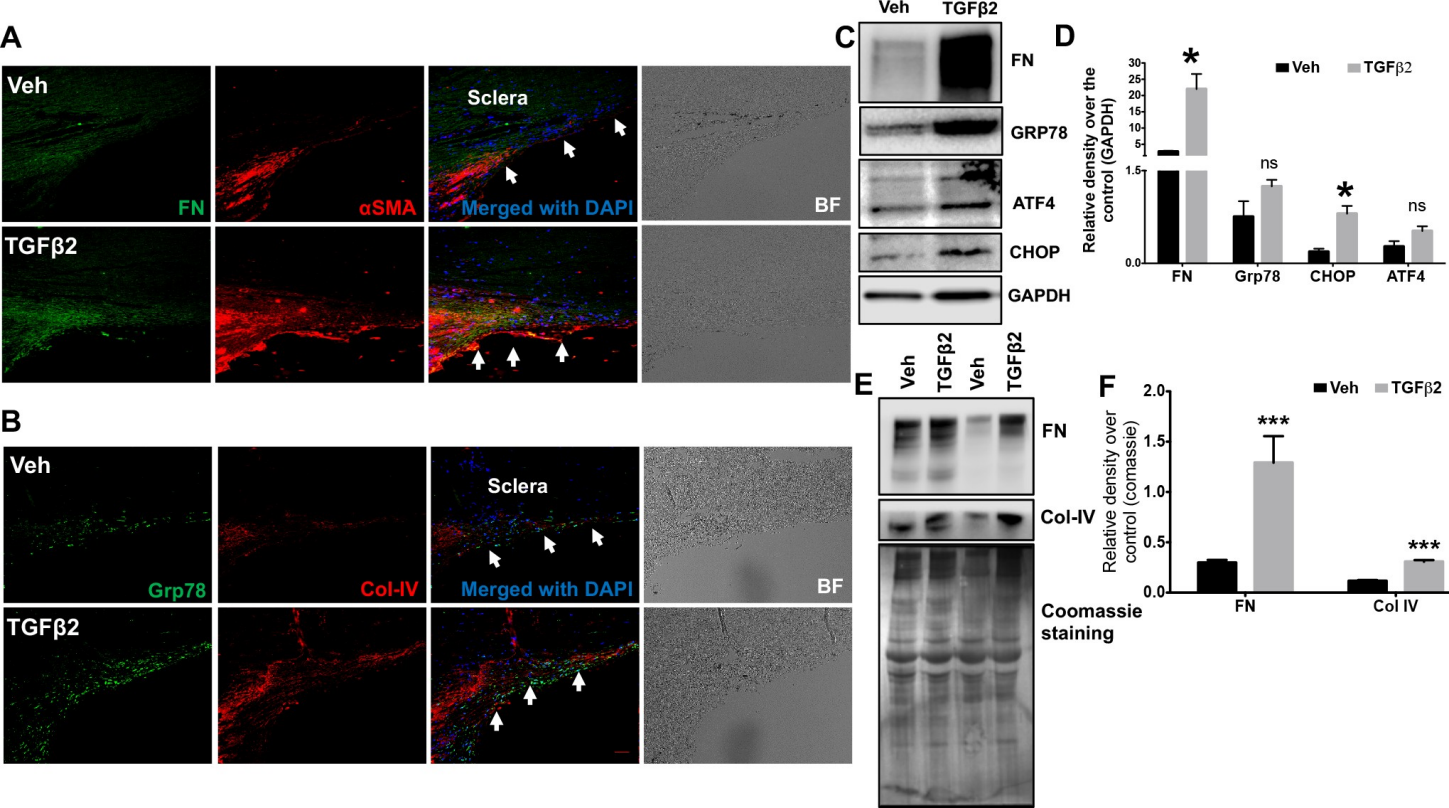
Increased ECM accumulation in the TM of TGFβ2-treated cultured corneoscleral segments. (**A** and **B**) Immunostaining for fibronectin (FN), collagen IV (Col-IV), αSMA and GRP78 in vehicle and TGFβ2 (5ng/ml) treated cultured corneoscleral segments. (n = 4 biological replicates, scale bar is 50μm). Western blot and densitometric analysis for FN, Col-IV (ECM markers), ATF4, CHOP, GRP78 in the TM tissue lysates (**C-D**) and conditioned medium (**E-F**) of vehicle and TGFβ2-treated cultured corneoscleral quadrants (n = 4 biological replicates for lysates and n = 8 for the conditioned medium), unpaired t-test, *P<0.05, **P<0.01, ***P<0.001. Arrows indicate the TM region.

**Fig 6:**
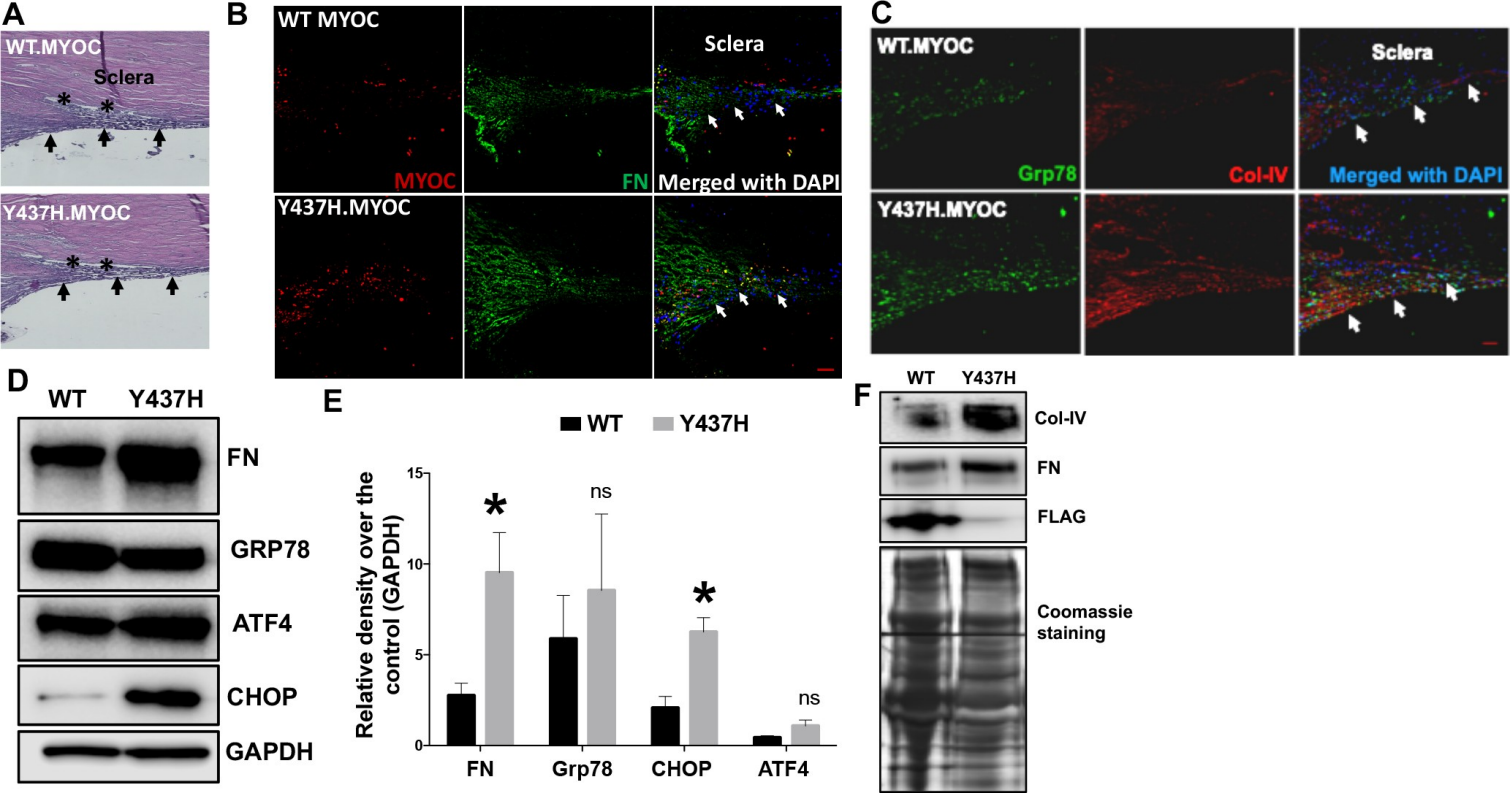
Lentiviral expression of mutant myocilin induces ER stress and ECM changes in the TM of cultured corneoscleral segments. The cultured corneoscleral quadrants were transduced with FTS tagged (FLAG & S tag) WT myocilin or mutant myocilin (Y437H) expressing lentiviral particles (1ml of lentivirus supernatant) for 7 days. **A**) H&E staining and (**B&C**) immunostaining for myocilin, FN, GRP78 and Col-IV in cultured corneoscleral segments transduced with WT or mutant myocilin. Increased myocilin staining was observed in the TM of mutant myocilin-transduced quadrants compared to WT myocilin. In addition, increased FN and Col-IV staining indicate more ECM accumulation in the TM of mutant myocilin-transduced quadrants (n = 3 biological replicates, scale bar is 50μm). Western blot and densitometric analysis of TM tissue lysates (**D-E**) and conditioned medium (**F**) obtained from cultured quadrants transduced with WT and mutant myocilin lentiviral expression vectors. A significant increase in the ECM marker FN (n = 6 biological replicates) and the ER stress marker CHOP (n = 3 biological replicates) was observed in mutant myocilin-transduced TM tissue lysates. Similarly, conditioned medium (**F**) from mutant myocilin-treated corneoscleral segments showed increases in ECM proteins FN and Col IV. Moreover, WT myocilin was detected in conditioned media of WT myocilin-transduced quadrants while no myocilin was detected in quadrants expressing mutant myocilin indicating that expression of mutant myocilin inhibits its secretion and accumulates in the TM cells. Unpaired t-test, *P<0.05, **P<0.01, ***P<0.001. Arrows indicate the TM region.
