# Supplementary material for: Ex-vivo cultured human corneoscleral segment model to study the effects of glaucoma factors on trabecular meshwork
Source: PLoS One. 2020 Jun 24;15(6):e0232111. doi: 10.1371/journal.pone.0232111 (PMC7314024; doi:10.1371/journal.pone.0232111)
Supplement: S1 Data — (PDF) [file pone.0232111.s001.pdf]

Fig.4C

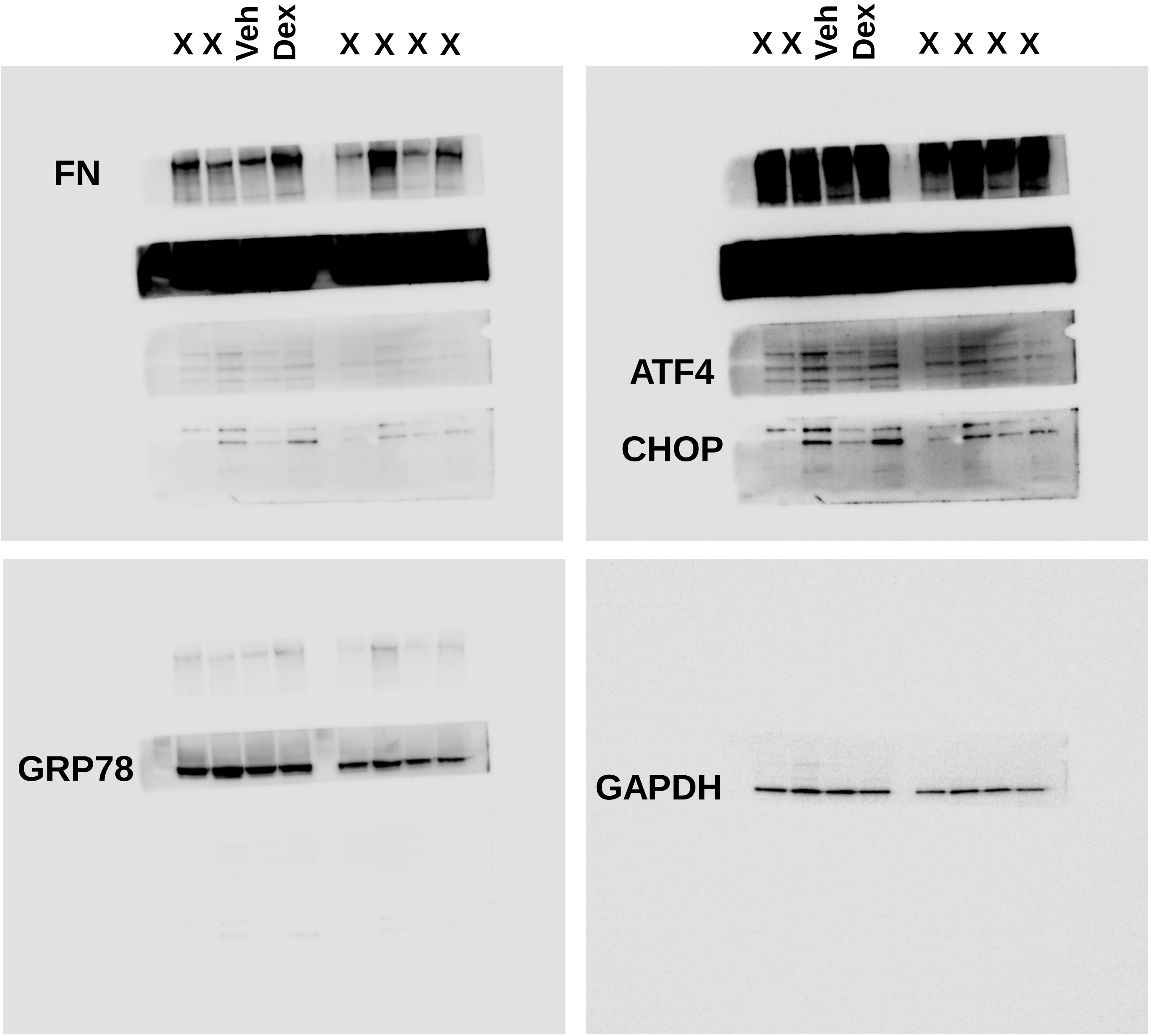

Fig.4E

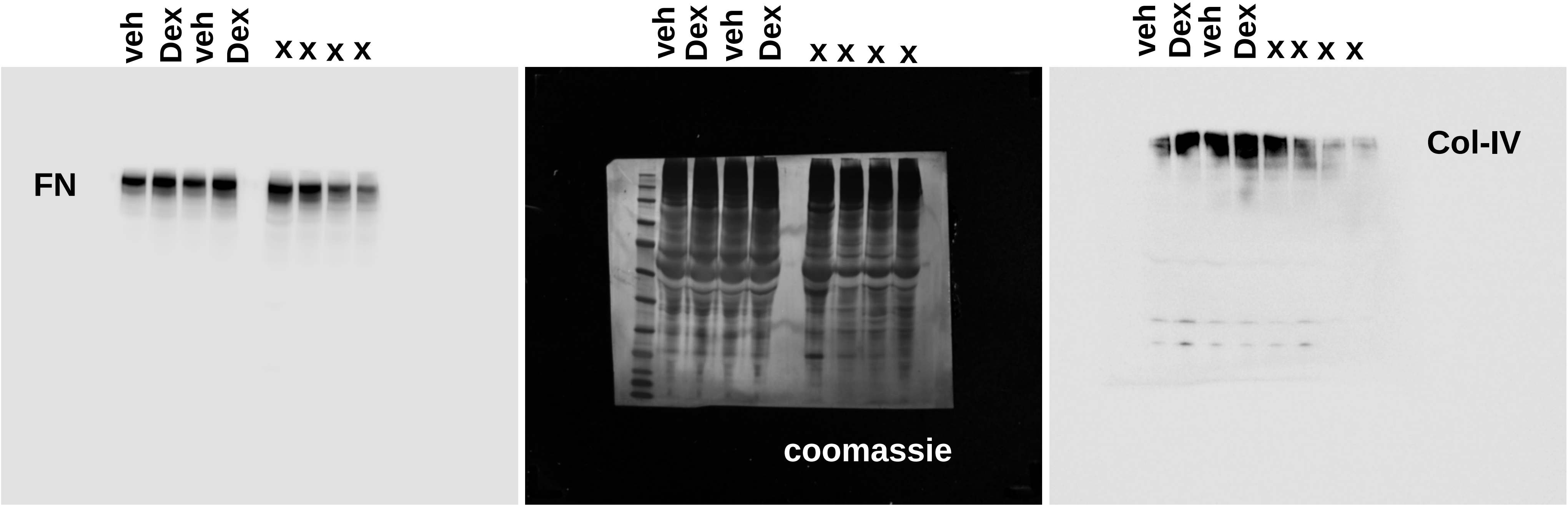

Note: We have loaded an equal amount of same samples on two different blots and stained one with FN and one with Col-IV. When we tried the same blot with both antibodies (FN and Col-IV , will give almost similar band sizes), one after the other after stripping the blot, the staining did not work.

Fig.5C

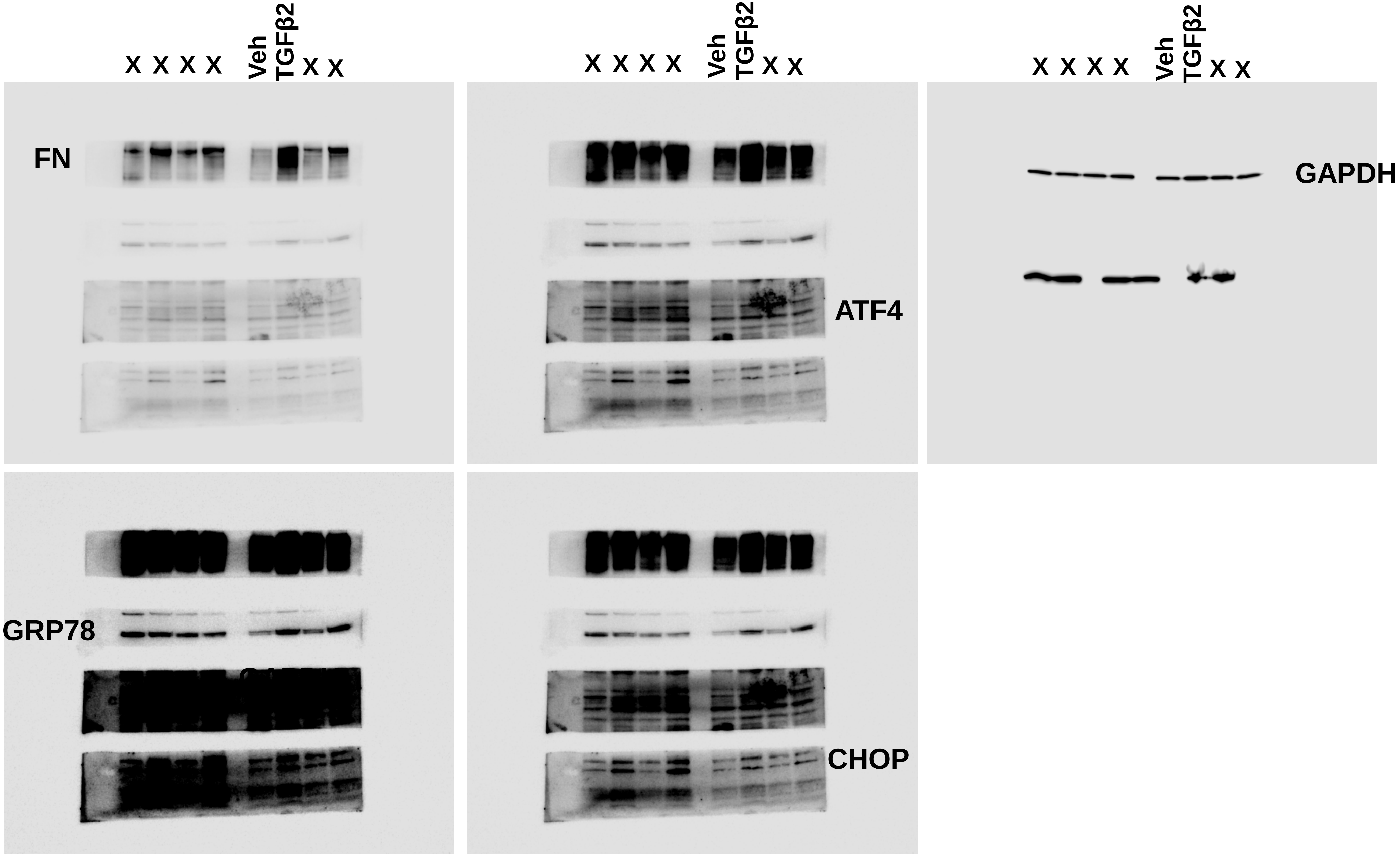
X X X X Veh TGFβ2 X X
X X X X Veh TGFβ2 X X
X X X X Veh TGFβ2 X X
FN
ATF4
GAPDH
GRP78
CHOP

Fig.5E

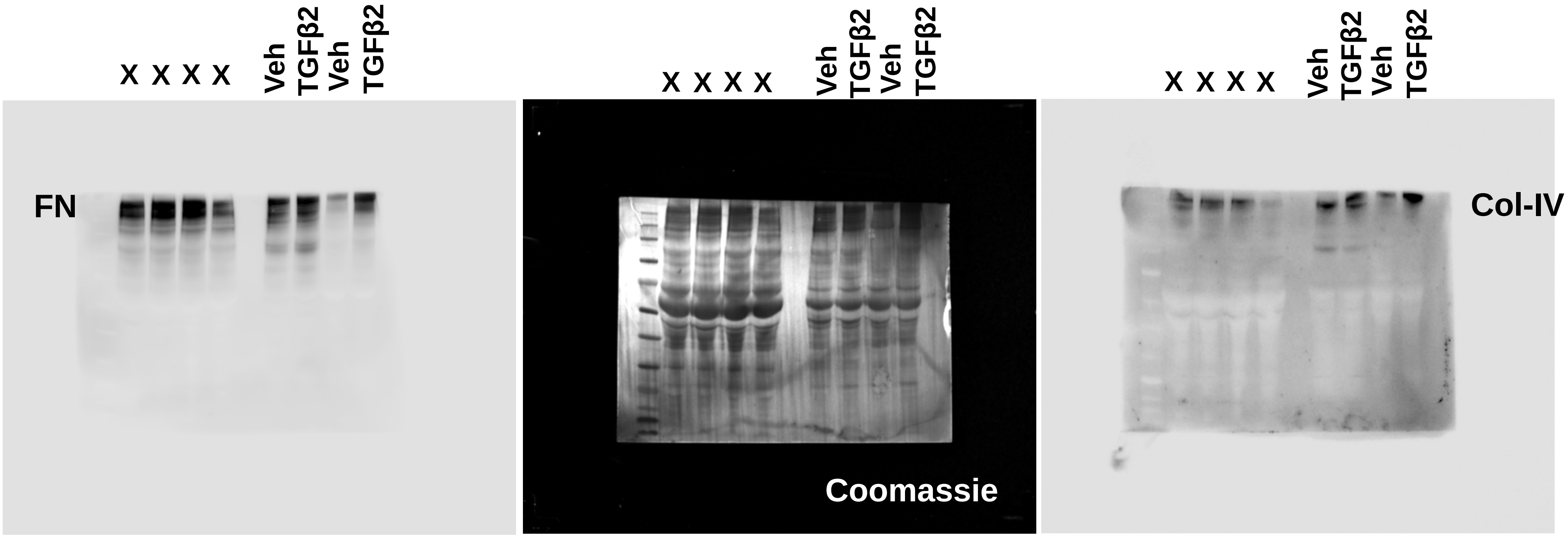
X X X X
Veh
TGFβ2
Veh
TGFβ2
FN
X X X X
Veh
TGFβ2
Veh
TGFβ2
Coomassie
X X X X
Veh
TGFβ2
Veh
TGFβ2
Col-IV

Fig.6D

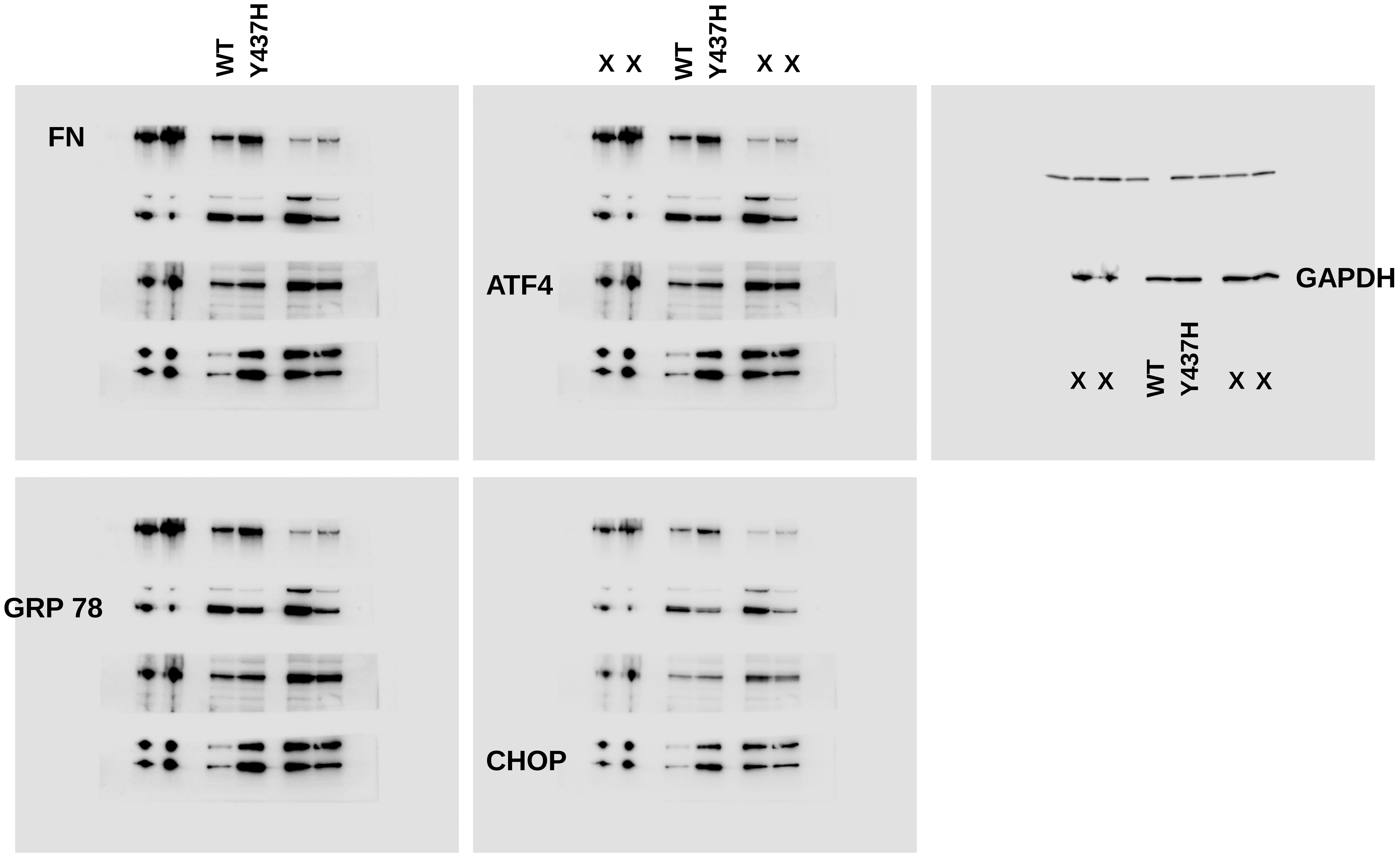

Fig.6F

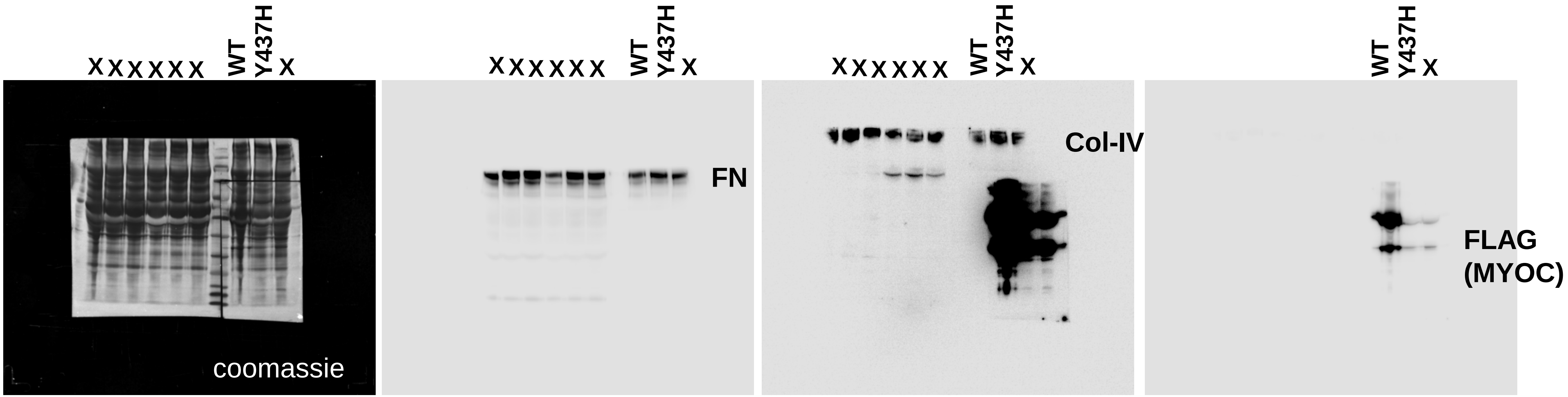

X X X X X X WT Y437H X
coomassie
X X X X X X WT Y437H X
FN
X X X X X X WT Y437H X
Col-IV
WT Y437H X
FLAG
(MYOC)
