# Supplementary material for: Ex-vivo cultured human corneoscleral segment model to study the effects of glaucoma factors on trabecular meshwork
Source: PLoS One. 2020 Jun 24;15(6):e0232111. doi: 10.1371/journal.pone.0232111 (PMC7314024; doi:10.1371/journal.pone.0232111)
Supplement: Attachment — Submitted filename: Response to Editor comments.docx [file pone.0232111.s002.docx]

**Editorial comments:**

We note that your study involved donated tissue/organs. Please provide the following information regarding tissue/organ donors for cases analyzed in your study.

1. Please provide the source(s) of the transplanted tissue/organs used in the study, including the institution name and a non-identifying description of the donor(s).

**Authors Response:** We have procured the **postmortem** human donor corneas from the Lions Eye Institute for Transplant and Research, Tampa. The table below describes non-identifying donor information.

| Tissue ID | Donor Age | Male/Female | OD/OS | Race |
| --- | --- | --- | --- | --- |
| 8762 | 59 | M | OS | White |
| 8763 | 59 | M | OD | White |
| 9436 | 67 | F | OS | White |
| 9437 | 67 | F | OD | White |
| 9450 | 57 | M | OS | Black |
| 9451 | 57 | M | OD | Black |
| 0658 | 76 | M | OD | White |
| 0659 | 76 | M | OS | White |
| 0242 | 65 | M | OD | White |
| 0243 | 65 | M | OS | White |
| 0244 | 62 | F | OD | White |
| 0245 | 62 | F | OS | White |
| 0286 | 69 | M | OD | White |
| 0287 | 69 | M | OS | White |
| 0179 | 76 | M | OD | Caucasian |
| 0179 | 76 | M | OS | Caucasian |
| 0252 | 54 | M | OD | Caucasian |
| 0252 | 54 | M | OS | Caucasian |
| 0262 | 69 | F | OD | Caucasian |
| 0262 | 69 | F | OS | Caucasian |
| 5017 | 41 | M | OS | Caucasian |
| 5017 | 41 | M | OD | Caucasian |
| 4983 | 29 | M | OS | Caucasian |
| 4983 | 29 | M | OD | Caucasian |
| 12252 | 73 | M | OD | Other |
| 12252 | 73 | M | OS | Other |
| 11848 | 59 | NP | OD | Caucasian |
| 11848 | 59 | NP | OS | Caucasian |
| 17292 | 68 | M | OD | Caucasian |
| 17292 | 68 | M | OS | Caucasian |
| 17331 | 59 | M | OD | Caucasian |
| 17331 | 59 | M | OS | Caucasian |
| 0508 | 60 | F | OD | Hispanic |
| 0508 | 60 | F | OS | Hispanic |
| 0612 | 68 | F | OD | Caucasian |
| 0612 | 68 | F | OS | Caucasian |

2. Please state in your response letter and ethics statement whether the transplant cases for this study involved any vulnerable populations; for example, tissue/organs from prisoners, subjects with reduced mental capacity due to illness or age, or minors.
- If a vulnerable population was used, please describe the population, justify the decision to use tissue/organ donations from this group, and clearly describe what measures were taken in the informed consent procedure to assure protection of the vulnerable group and avoid coercion. If a vulnerable population was not used, please state in your ethics statement, “None of the transplant donors was from a vulnerable population and all donors or next of kin provided written informed consent that was freely given.”

**Authors response:** None of the transplant donors was from a vulnerable population and all donors or next of kin provided written informed consent that was freely given.

3. In the Methods, please provide detailed information about the procedure by which informed consent was obtained from organ/tissue donors or their next of kin. In addition, please provide a blank example of the form used to obtain consent from donors, and an English translation if the original is in a different language.

**Authors response:** The Lions Eye Institute for Transplant and Research (LEITR) is registered under the Eye Bank Association of America (EBAA), and it follows EBAA regulations to obtain donor tissues. We directly procure the deidentified postmortem tissues from LEITR, which obtains the required informed consent. We have contacted LEITR to provide further information regarding the informed consent obtained and tissue procurement procedure.

4. Please state whether the donors specifically provided consent for the use of their donated tissue for both transplantation and donation purposes.

**Authors response:** Consent is obtained from donors by Lions Eye Bank.

5. Please indicate whether the donors were previously registered as organ donors. If tissues/organs were obtained from deceased donors or cadavers, please provide details as to the donors’ cause(s) of death.

**Authors response:** All tissues were obtained from organ donors.

6. Please provide the participant recruitment dates and the period during which transplant procedures were done (as month and year).

**Authors response:** Not applicable.

7. Please discuss whether medical costs were covered or other cash payments were provided to the family of the donor. If so, please specify the value of this support (in local currency and equivalent to U.S. dollars).

**Authors response:** Not applicable.

8. Please state whether you had access to any identifying information about the donors (e.g. names/addresses) as part of this study."

**Authors response:** We do not have access to identifying donor information. We have received only the non-identifying donor information sheets along with rejected donor corneas.

3.  PLOS ONE now requires that authors provide the original uncropped and unadjusted images underlying all blot or gel results reported in a submission’s figures or Supporting Information files. This policy and the journal’s other requirements for blot/gel reporting and figure preparation are described in detail at https://journals.plos.org/plosone/s/figures#loc-blot-and-gel-reporting-requirements and https://journals.plos.org/plosone/s/figures#loc-preparing-figures-from-image-files. When you submit your revised manuscript, please ensure that your figures adhere fully to these guidelines and provide the original underlying images for all blot or gel data reported in your submission. See the following link for instructions on providing the original image data: https://journals.plos.org/plosone/s/figures#loc-original-images-for-blots-and-gels.

In your cover letter, please note whether your blot/gel image data are in Supporting Information or posted at a public data repository, provide the repository URL if relevant, and provide specific details as to which raw blot/gel images, if any, are not available. Email us at plosone@plos.org if you have any questions.

**Authors response:** We have provided original uncropped and unadjusted images of all blots and gels reported in the manuscript figures as per the journal guidelines.

4. We note that you have included the phrase “data not shown” in your manuscript. Unfortunately, this does not meet our data sharing requirements. PLOS does not permit references to inaccessible data. We require that authors provide all relevant data within the paper, Supporting Information files, or in an acceptable, public repository. Please add a citation to support this phrase or upload the data that corresponds with these findings to a stable repository (such as Figshare or Dryad) and provide and URLs, DOIs, or accession numbers that may be used to access these data. Or, if the data are not a core part of the research being presented in your study, we ask that you remove the phrase that refers to these data.

**Authors response:** We have removed the sentence “data not shown” and also the phrase that refers to this data.
