# Supplementary material for: Ex-vivo cultured human corneoscleral segment model to study the effects of glaucoma factors on trabecular meshwork
Source: PLoS One. 2020 Jun 24;15(6):e0232111. doi: 10.1371/journal.pone.0232111 (PMC7314024; doi:10.1371/journal.pone.0232111)
Supplement: Attachment — Submitted filename: Authors response Kasetti Plos 040320.docx [file pone.0232111.s003.docx]

**Authors Response Letter**

We appreciate the reviewers’ valuable insights and suggestions which have substantially improved our manuscript. We have carefully addressed all concerns and performed additional experiments to address these concerns. These changes are highlighted in blue in the revised manuscript. A detailed response to the reviewer’s concerns is provided below:

1. GRP78 is not significant under any of the treatments in the study as measured by Western blot. This needs to be discussed or more experiments need to be conducted to investigate this.

**Authors response:** We agree with the reviewer’s comment and are thankful for their suggestion. Although there is a trend showing an increase in GRP78 expression in response to treatment with glaucoma factors, this increase is not significant in Western blot analysis. Please note that immunostaining for GRP78 as shown in Figures 4B, 5B and 6C clearly demonstrate that glaucoma factors increase GRP78 staining in the TM. We have added this clarification in the results and further included the following explanation in the discussion:

“We observed that glaucoma causing factors significantly increased chronic ER stress markers, CHOP and ATF4 but not GRP78 in the TM tissues of corneoscleral segments. Although GRP78 expression showed an increasing trend, the difference compared to control was not significant. GRP78 is a key activator of ER stress sensors (IRE1, PERK and ATF6) that activates the UPR pathway. GRP78 is normally bound to these ER stress sensors, thus keeping them inactivated. Under stress conditions or accumulation of misfolded proteins, GRP78 is released from these sensors, resulting into activation of ER stress sensors and UPR pathway [1]. Since GRP78 is expressed abundantly in TM tissues, a small increase after treatment with glaucoma factors may be sufficient to activate the UPR pathway. This is apparent from significantly increased ATF4 and CHOP, which are downstream of GRP78 and are considered classical markers of chronic ER stress.”

2. Under Dex and mutant MYOC treatments, cellular fibronectin is not significant, even though the secreted form is. This needs to be addressed in the discussion, or more experiments conducted to investigate.

**Authors response:** We thank the reviewer for their suggestion. In the present study, we observed differences in Dex response between donor eyes. Dex treatment-induced ECM changes were observed in ~50% of the treated corneoscleral segments. In our previous version of this manuscript, we included data from the non-responders as well as Dex responders for densitometric analysis. We re-analyzed the data using only the Dex responders and observed a significant increase in FN expression in the lysates (Figure 4D). For the mutant MYOC treatments, 3 additional biological replicates were run and analyzed to show significant change in FN expression (Figure 6E). We have edited the results and figures to include this new data.

3. Could the authors discuss why they investigated secreted ColIV in conditioned media and not the cellular form by Western blot? I’d like to see cellular ColIV measured by Western blot for comparison.

**Authors response:** We agree with the reviewer’s comment and appreciate their constructive feedback. The TM tissue peeled from each quadrant is only sufficient to run a single Western blot. Furthermore, the ColIV antibody did not work on the stripped blots that were previously probed with FN antibody since the band sizes for both FN and ColIV fall in the same vicinity. This is why we were only able to probe for FN in lysates, whereas FN and ColIV both were analyzed in the conditioned medium, which was available in sufficient quantities for multiple Western blots.

4. In Fig 6B. there is no visible MYOC present in the indicated TM region under the lentiviral treatment. This does not support the author’s statements regarding accumulated cellular mutant MYOC in TM cells.

**Authors response:** We thank the reviewer for their comment. We have now revised Figure 6B with new images, which clearly show increased mutant myocilin accumulation compared to WT MYOC.

5. Finally, the sentence regarding comparison of the TM structural integrity of these corneoscleral quadrants to intact human eyes is still included the discussion

**Authors response:** We sincerely regret this oversight and as per the reviewer’s suggestion, we have made the correction by removing the statement from the discussion (Page 21, line 5) and replaced with the following statement. Note that we mentioned TM morphology since we examined TM morphology via H&E staining.

“Despite this, we did not observe any notable changes in the TM morphology in these corneoscleral segments.”

References:

1.	Roussel BD, Kruppa AJ, Miranda E, Crowther DC, Lomas DA, Marciniak SJ. Endoplasmic reticulum dysfunction in neurological disease. Lancet Neurol. 2013;12(1):105-18. Epub 2012/12/15. doi: 10.1016/S1474-4422(12)70238-7. PubMed PMID: 23237905.
